# Long-Lasting Verbatim Memory for the Words of Books After a Single Reading Without Any Learning Intention

**DOI:** 10.3389/fpsyg.2020.01780

**Published:** 2020-07-24

**Authors:** Christof Kuhbandner

**Affiliations:** Department of Psychology, University of Regensburg, Regensburg, Germany

**Keywords:** text learning, verbatim memory, recognition without awareness, perceptual memory, visual memory

## Abstract

The present study reveals an intriguing ability of our human memory: when reading a book once without any intention of learning, we store long-lasting verbatim memories of the words written in the book without being aware of it. Participants read a book chapter consisting of 32 pages (3,772 words) once without knowing that their memory would be tested later. In memory tests immediately after reading and 1 week after reading, they were asked to remember exactly which word was written at a specific position in the book chapter. Only memory for words was tested that were theme-unrelated and non-central. To measure memory, a two-alternative forced choice recognition test was used where a page was shown either as read before or with the replacement of one single word by a synonym. For each response, participants indicated whether the response was based on phenomenal memory experience (recollection or familiarity) or guessing. In the immediate test, participants claimed to have phenomenal memory experience for about a quarter of the tested positions, truly remembering the word in about half of cases. In the 1-week-delayed test, phenomenal memory experience was nearly entirely absent and completely uninformative. When claiming to have no phenomenal memory experience, participants still truly remembered the word for about 10% of the tested positions in both the immediate test and the 1-week-delayed test, without any forgetting. These findings demonstrate that we store more from read texts in memory than commonly believed.

## Introduction

According to numerous articles, books, and documentary films, he was one of the greatest memory giants, who has ever lived: Kim Peek, a savant who displayed phenomenal memory skills. Probably most fascinating was his ability to memorize books verbatim after reading them once. For instance, according to an article by the savant-researchers Treffert and Christensen (2005), he read Tom Clancy’s The Hunt for Red October – a book with 656 pages in paperback version – in 1 h and 25 min. When asked 4 months later, he was able to give the name of the Russian radio operator in the book, referring to the page describing the character and quoting several passages verbatim. When hearing about the abilities of savants like Kim Peek, we commonly conclude that such memory skills are highly exceptional. The aim of this study was to examine whether it may be that actually we all store long-term verbatim memory representations of the words written in books that we have read once without any intention of learning.

At first glance, the existence of verbatim long-term memory representations for the words written in read books seems highly unlikely. From an introspective perspective, after having read a book, we seem unable to remember exactly which word was written at a specific position in the book. In fact, this is the reason why the memory skills of savants like Kim Peek seem to be so unbelievable and thus highly exceptional. Consistent with this everyday intuition, many studies have shown that people seem to forget the surface details of read texts as soon as the content has been understood, and to store only the underlying abstract meanings (the so-called “gist”; e.g., Anderson, 1974; Johnson-Laird et al., 1974; Gernsbacher, 1985). Based on such findings, conventional wisdom in memory research is that only abstract representations of the meanings of read texts are stored in long-term memory, whereas the exact words of a text are quickly forgotten and not stored in long-term memory (e.g., Loebell and Bock, 2003; Christiansen and Chater, 2016).

However, to achieve progress in the understanding of human memory, it is important to challenge existing beliefs, even if they seem close to being trivially true. Interestingly, recent studies have shown that humans store much more information in long-term memory than commonly believed. For instance, it has been demonstrated that observers can recognize details of thousands of pictures after having studied them only for a few seconds each (Brady et al., 2008; Konkle et al., 2010). Even more intriguingly, subsequent research has shown that long-lasting detailed long-term memories are even stored when stimuli are presented only for 500 ms and processed without any attention and intention of learning (Kuhbandner et al., 2017; Hutmacher and Kuhbandner, 2020).

Importantly, as shown in the latter studies and other related studies in the field, people seem often not to be aware of their memory abilities. For instance, in the study on long-lasting memory for unattended and briefly presented pictures, memory was tested 24 h after picture presentation with a two-alternative forced choice test, where a previously seen picture was paired with a foil picture that had not been presented before. Although the participants claimed to have no phenomenal memory experience (recollection or familiarity) and thus to guess on 94.5% the memory test trials, they still remembered 14.1% of the previously presented objects correctly (corrected for guessing).

The finding that performance can be above chance in n-alternative forced choice recognition test despite participants claiming that they are guessing without having any phenomenal memory experience is supported by several studies (e.g., Voss et al., 2008; Craik et al., 2015; Hutmacher and Kuhbandner, 2018). Based on such findings, it has been assumed that there is a third subtype of memory beyond the awareness-dependent (explicit) subtypes of recollection and familiarity: an unconscious (implicit) subtype of memory, which is not accompanied by a phenomenal (i.e., conscious) experience of remembering, termed “recognition without awareness” or “implicit recognition” (for a review, see Voss et al., 2012). Such an implicit subtype of memory is also well supported by studies on the so-called repetition priming effect, demonstrating that a brief exposure to a visual picture without mentioning that memory will be tested later leads to a processing benefit when the picture is encountered again after several days, months, or even years, with participants often being unaware of their memory abilities (Mitchell and Brown, 1988; Mitchell, 2006; Larzabal et al., 2018; Mitchell et al., 2018).

Thus, if a study aims to examine whether verbatim long-term memory representations are stored for the words written in read books, it is important to use memory tests that are sensitive enough to also measure memory representations that are below the level of phenomenal memory awareness. Regarding classic types of memory test, it is important to note that recognition without awareness can only occur in n-alternative forced choice tests where participants are asked to choose the previously encountered (i.e., old) item among new items. As participants are forced to choose an item even when having neither a phenomenal experience of recollection nor a phenomenal experience of familiarity, they can choose the old item driven by unconscious memory processes. In an old-new recognition test where participants are shown old and new items on separate trials with the instruction to indicate for each individual item whether it is old or new, when having no phenomenal memory experience, participants choose the response option “new”, although they may actually implicitly remember the item. Consequently, if the aim is to measure stored memory representations as sensitively as possible, it is important to measure memory with n-alternative forced choice tests because recognition without awareness can only occur in such tests. This is supported by studies showing that performance in two-alternative forced choice recognition tests is substantially higher than performance in old-new recognition tests (e.g., Cunningham et al., 2015).

Taken together, it may indeed be possible that long-lasting verbatim representations of the words of a read book are stored in long-term memory despite the subjective phenomenal experience of absent memory. The reason why such representations have not been found in previous research may be that memory tests were used that were not sensitive enough to detect them. First preliminary evidence that readers maintain something from solely read texts in long-term memory comes from a study showing that a typographically inverted text that has been read 1 year ago is read faster than a typographically inverted text that has not been read before (Kolers, 1976). Further preliminary evidence comes from a recent study showing that the surface syntax of text passages that have been heard once without any intention of learning can be recognized above chance levels and is even freely reproduced when asked to report what has been heard (Gurevich et al., 2010). However, only relatively short texts were used in these studies so that it remains to be shown whether this memory ability generalizes to the reading of whole books. In particular, it is unknown whether people are not only able to remember the syntax but also the exact words that were written at specific positions in the book.

**Figure 1:**
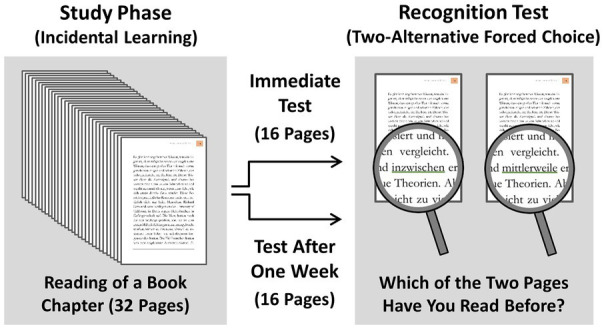
Memory paradigm. Participants read a book chapter consisting of 32 pages (3,772 words) one single time on their own pace with the instruction to judge the didactic quality of the book chapter afterwards. No mention was made that their memory would be tested later. In a surprise memory test, verbatim memory for the book chapter was tested by asking the participants to remember exactly which word was written at a specific position on a page in the book. A two-alternative forced choice recognition test was used where a page was shown either as read before or with the replacement of one single theme-unrelated and non-central word by a synonym of the same length. For half of the pages, memory was tested immediately after reading, for the other half, memory was tested after a delay of 1 week.

The present study examined this by asking participants to read a book chapter consisting of 32 pages (3,772 words) with the instruction to evaluate the didactic quality. No mention was made that their memory would be tested later. In a surprise memory test, their verbatim memory for the words written in the book chapter was tested by asking them to remember exactly which word was presented at a specific position in the book. To rule out the possibility that the observed memory performance may only reflect memory for central words that are relevant for the theme of the chapter, only memory for words was tested that were theme-unrelated and non-central (for examples, see Table 1). To measure memory representations that are not necessarily accompanied by the phenomenal memory experiences of recollection or familiarity, a two-alternative forced choice recognition test was used where a page was shown either as read before or with replacement of one single word by a synonym of the same length (for an illustration, see Figure 1).

To convey the existence of recognition without awareness, participants were told that they will probably have the feeling of not knowing the answer in many cases, but that previous studies have shown that participants can nevertheless perform remarkably well in such situations when they base their decisions on their intuition. Previous research has shown that instructing participants in such a way is a precondition to sensitively measure recognition without awareness (Voss and Paller, 2010). To measure participants’ subjective memory awareness, for each response in the test, participants were asked to indicate whether the response was based on the phenomenal memory experience of knowing what had been read before, or whether they had guessed. To examine the durability of stored memories, for half of the pages, memory was tested immediately after reading, and for the other half of the pages, memory was tested after a delay of 1 week.

## Materials and Methods

### Participants

The experiment was preregistered^1^. A power analysis (G*Power 3.1.718; Faul et al., 2007) yielded a target sample size of 41 to have sufficient power (0.80, alpha = 0.05, one-tailed) in order to detect small-to-medium sized effects (*d* = 0.4). Overall, 43 undergraduate students participated for course credit. Data from two participants had to be excluded because they went through the pages of the book without reading (one participant skipped all pages with a mean reading time per skipped page of 0.28 s, the other participant skipped 16% of the pages with a mean reading time per skipped page of 2.95 s; including the remaining data of the latter participant did not change any of the significant results). These participants were replaced in order to reach the planned sample size of 41 participants (34 females; mean age = 22.6 years, *SD* = 2.9). The study was conducted in accordance with the Helsinki Declaration and the University Research Ethics Standards. In Germany, these types of psychological studies do not require ethical approval of an Ethics Committee (see https://www.dfg.de/foerderung/faq/geistes_sozialwissenschaften/). All participants provided written informed consent. All data exclusions, all manipulations, and all measures in the study are reported.

### Materials and Procedure

The procedure of the study involved an initial reading phase, an immediate memory test that was conducted directly after reading, and a delayed memory test that was conducted after 1 week. In the initial reading phase, all participants received an ebook reader that contained 32 pages (3,772 words) of Michael Gazzaniga’s book *Who’s in Charge?: Free Will and the Science of the Brain* (Gazzaniga, 2012; German language edition). Two versions of the 32 pages were prepared. One version corresponded to the pages of the original book. In the other version, on each of the pages, one single word was replaced by a synonym of the same length. The replaced word was non-central and irrelevant for the theme of the chapter. Examples of sentences with replacement of one single non-central word are shown in Table 1 (the whole material can be downloaded at https://osf.io/g3ju6/?view_only=1165978bffeb441ead9e1380574f8de2).

**Table 1:** Example sentences with the replacement of one single non-central word (printed in bold).

Version A	Version B	English translation
Es hat Hunderte von Jahren gedauert, unser **gegenwärtiges** Wissen über den Aufbau des menschlichen Gehirns zu gewinnen, und es war kein einfacher Weg.	Es hat Hunderte von Jahren gedauert, unser **momentanes** Wissen über den Aufbau des menschlichen Gehirns zu gewinnen, und es war kein einfacher Weg.	It has taken hundreds of years to establish our **current** knowledge of the structure of the human brain, and it has not been an easy path.			
Einige seiner Geheimnisse sind **inzwischen** geklärt, und ständig entstehen neue Theorien.	Einige seiner Geheimnisse sind **mittlerweile** geklärt, und ständig entstehen neue Theorien.	Some of his mysteries have **now** been clarified, and new theories are constantly emerging.			
Es gibt also hochspezialisierte Module, in diesem Fall zur Identifikation, die weder Erfahrung noch sozialen Kontext **brauchen**, um zu funktionieren.	Es gibt also hochspezialisierte Module, in diesem Fall zur Identifikation, die weder Erfahrung noch sozialen Kontext **benötigen**, um zu funktionieren.	There are thus highly specialized modules, in this case for identification, that **need** neither experience nor a social context in order to function.

Note: On each page of the book, only one single word was replaced by a synonym.

Which version was initially read was counterbalanced across participants. They were asked to read the 32 pages once at their own pace with the instruction to judge the didactic quality of the book chapter after reading (Instruction: “Your task: this study is about evaluating the quality of a textbook. For this purpose, you will read an approximately 30-page chapter of the textbook. Please read the text carefully from top to bottom at your own pace”). They were not allowed to take any notes. No mention was made that their memory would be tested later. To prevent the scrolling back to previous pages, it was not possible to move back to the previous page after having moved to the next page. Before analyzing any results, for each participant, the reading time for each page was determined. Pages that were viewed so shortly that the reading of individual words was impossible were excluded (*M*_Reading Time_ = 39.9 s, *SD* = 12.8; min-max = 13.4–108.7; 3 out of 1,312 pages were excluded, with reading times of 0.9, 1.4, and 1.5 s).

After finishing reading, the ebook reader was handed to the experimenter, and the participants were asked to move to a computer in order to take a surprise memory test. In both the immediate memory test and the 1-week-delayed memory test, verbatim memory for the contents of the book was tested by a two-alternative forced choice recognition test. On each test trial, the two versions of a page (original version and version with replacement of one single word by a synonym) were shown on a computer screen. Consequently, one version was identical to the page read before, and the other version differed from the page read before only in one non-central word that was replaced by a synonym of the same length. On half of the recognition test trials, the previously read page was shown on the left side, in the other half on the right side; the order was randomized across test trials. To facilitate the finding of the word that differed between the two pages, the original word and the synonym were underlined in green in the memory test (for an illustration, see Figure 1). Participants were instructed to indicate which of the two page versions they had read before. Participants were told that this task will not be easy, and that they will probably have the feeling of not knowing the answer in many cases. They were also told that numerous previous studies have shown that participants can nevertheless perform remarkably well in such situations when they base their decisions on their intuition, and they were asked to follow their “gut feelings” when not knowing an answer. Previous research has shown that such a memory test measures the amount of stored information more sensitively than when participants base their memory responses on experiences of recollection and familiarity (Voss and Paller, 2010).

In the immediate memory test directly after reading, memory for the first half of the pages (pages 1–16) was tested. In the memory test after 1 week, memory for all pages was tested. Memory for the pages that had not been tested in the immediate test were tested first (pages 17–32). In both memory tests, the order of testing followed the order of initial reading, and participants were allowed to proceed at their own pace. After each memory response, participants were asked to indicate whether they have known which page they had read before or guessed. After the immediate test, participants were asked to report whether they had expected that their memory for the book chapter would be tested later. None of the participants expected a later memory text.

### Statistical Analysis

As a correct response in a two-alternative forced choice test can reflect not only a true memory response but also a fortunate guess, to estimate the true percentage of remembered words (PR_True_), the observed percentage correct (PC_Observed_) has to be corrected for fortunate guesses (formula: PR_True_ = 2 × PC_Observed_ – 100; e.g., Brady et al., 2013). To determine whether memory performance was above chance (i.e., a corrected memory performance of PR_True_ = 0), one-sample *t*-tests with one-tailed alpha level were used for data analysis.

## Results

### Overall Memory Performance

**Figure 2:**
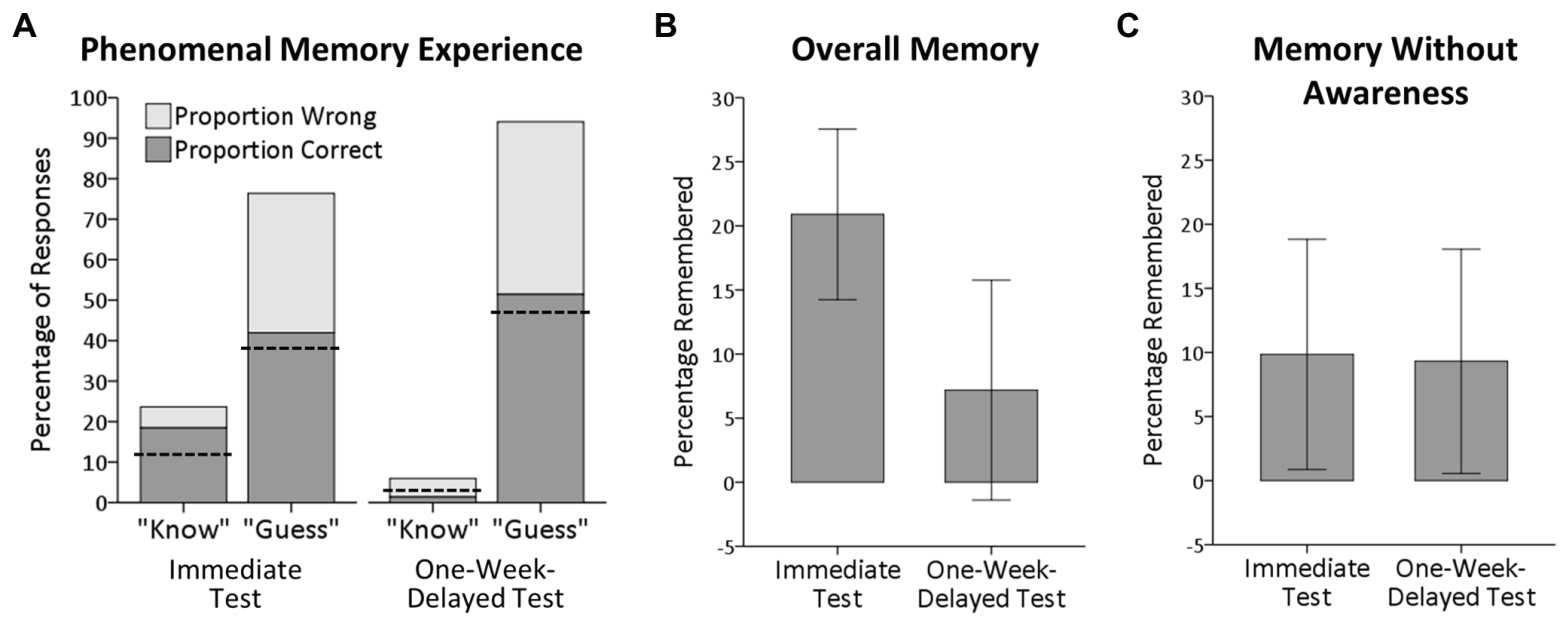
**(A)** Phenomenal memory experience and objective memory performance. The total height of the bars shows the percentage of tested positions for which participants claimed to have (“know” responses) or have no (“guess” responses) phenomenal memory experience in the immediate and the 1-week-delayed memory tests. The dark gray bars show the proportion of correct memory responses (choosing the previously read page) and the light gray bars show the proportion of wrong memory responses (choosing the page with a word replaced by a synonym). The dashed lines indicate chance performance. **(B)** Overall memory performance. The height of the bars shows the percentages of words that were overall remembered (corrected for fortunate guesses) in the immediate and the 1-week-delayed memory tests, independently of participants’ phenomenal memory experience. **(C)** Memory without awareness. The height of the bars shows the percentages of words that were remembered on memory test trials where participants reported to have no phenomenal memory experience (“guess” trials) in the immediate and the 1-week-delayed memory tests (corrected for fortunate guesses). Error bars represent one-tailed 95% confidence intervals.

Figure 2A shows the reported phenomenal memory experience (percentage of “know” versus “guess” responses) and the observed objective performance on memory test trials where participants claimed to have (“know”) versus have no (“guess”) phenomenal memory experience in the immediate and 1-week-delayed memory tests. Figure 2B shows the percentage of words (corrected for guessing) that were overall remembered in the immediate and 1-week-delayed memory tests, independently of participants’ reported phenomenal memory experience.

In the immediate memory test, the percentage of overall remembered words was far above chance, *M*_PRTrue_ = 20.9%, *SD* = 25.3, one-tailed 95% CI [14.2, 27.5], *t*(40) = 5.29, *p* < 0.001 (one-tailed), *d* = 0.79. In the 1-week-delayed memory test, the percentage of overall remembered words for pages that were tested for the first time was slightly above chance, *M*_PRTrue_ = 7.2%, *SD* = 32.6, one-tailed 95% CI [−1.4, 15.8], *t*(40) = 1.41, *p* = 0.083 (one-tailed), *d* = 0.21. The percentage of overall remembered words for pages that had already been tested in the immediate test was slightly above chance as well, *M*_PRTrue_ = 7.4%, *SD* = 29.7, one-tailed 95% CI [−0.4, 15.2], *t*(40) = 1.60, *p* = 0.059 (one-tailed), *d* = 0.21, and did not differ from memory for pages that were tested for the first time, *t*(40) = −0.04, *p* = 0.968, *d* = 0.004. Combined across all pages, the percentage of overall remembered words in the 1-week-delayed memory test was significantly above chance, *M*_PRTrue_ = 7.3%, *SD* = 25.0, one-tailed 95% CI [0.6, 13.8], *t*(40) = 1.86, *p* = 0.035 (one-tailed), *d* = 0.27.

### Memory With Awareness

In the immediate memory test, participants reported to have phenomenal memory experience for 23.6% of the tested positions (*SD* = 18.5, 95% CI [70.5, 82.2]). In the 1-week-delayed test, participants reported to have phenomenal memory experience for 5.9% of the tested positions (*SD* = 12.3, 95% CI [70.5, 82.2]). The decrease in phenomenal memory experience was significant, *t*(40) = 7.59, *p* < 0.001, *d* = 1.19. Examining performance on memory test trials where participants reported to have a phenomenal memory experience and to have based their response on it (“know” responses) revealed that only about three out of four responses driven by phenomenal memory experience were correct in the immediate memory test, *M*_PCObserved_ = 78.0%, *SD* = 23.4, one-tailed 95% CI [71.1, 84.8]. Memory performance was far above chance (i.e., 50% correct), *t*(32) = 6.88, *p* < 0.001 (one-tailed), *d* = 0.94, but also far away from being perfect (i.e., 100% correct), *t*(32) = −5.42, *p* < 0.001 (one-tailed), *d* = 0.94. Assuming that some of the observed correct responses reflected fortunate guesses on trials where the phenomenal memory experience was not informative for the true memory ability, and correcting for such fortunate guesses, revealed that only on about half of the memory test trials where participants reported to have phenomenal memory experience the word at the tested position was truly remembered, *M*_PRTrue_ = 55.9%, *SD* = 46.7.

Since the number of “know” responses in the 1-week-delayed memory test was very small (*n* = 39 across all participants), with 68% of the participants reporting that they had no phenomenal memory experience at all, the reliability of participants’ phenomenal memory experience could not reliably be estimated. The data at least suggest that the reliability was much lower after 1 week since the percentage of correct responses on “know” trials was descriptively below chance performance, *M*_PCObserved_ = 23.5%, *SD* = 28.5, one-tailed 95% CI [9.2, 37.3].

### Memory Without Awareness

Figure 2C shows the percentage of remembered words (corrected for guessing) on memory test trials where participants reported to have no phenomenal memory experience (“guess” responses) in the immediate and 1-week-delayed memory tests. The percentage of remembered words on trials without phenomenal memory experience was significantly above chance both in the immediate memory test, *M*_PRTrue_ = 9.8%, *SD* = 34.2, one-tailed 95% CI [0.9, 18.8], *t*(40) = 1.85, *p* = 0.036 (one-tailed), *d* = 0.28, and the 1-week-delayed memory test, *M*_PRTrue_ = 9.3%, *SD* = 33.3, one-tailed 95% CI [0.6, 18.1], *t*(40) = 1.79, *p* = 0.040 (one-tailed), *d* = 0.27. Comparing memory without awareness between the immediate and 1-week-delayed tests revealed no difference, *t*(40) = 0.08, *p* = 0.938, *d* = 0.02. Data can be downloaded at https://osf.io/g3ju6/?view_only=1165978bffeb441ead9e1380574f8de2.

### Reading Time and Memory Performance

Finally, it was examined whether memory performance varied as a function of reading time. Reading time and memory performance were uncorrelated, both regarding overall memory performance in the immediate and delayed tests (*r*_Immediate_ = 0.01, *p* = 0.928; *r*_Delayed_ = 0.07, *p* = 0.670) and regarding memory without awareness in the immediate test and the delayed test (*r*_Immediate_ = 0.03, *p* = 0.839; *r*_Delayed_ = 0.06, *p* = 0.710).

## Discussion

The present study reveals an intriguing ability of human memory: we store long-lasting verbatim memories of the words of read books without being aware of it. When being asked to remember exactly which word was written at specific positions in a book chapter that was read 1 week before one single time without any intention of learning, participants stated on nearly every memory test trial that they have no phenomenal memory experience and thus have to guess. However, despite the absence of introspective memory awareness, participants still were able to remember for about 10% of the tested positions in the book chapter exactly which word was written at that position. This is the more astounding since memory for words was tested that were theme-unrelated and non-central. Comparing the percentages of words that were remembered verbatim without awareness immediately after reading and 1 week after reading revealed no forgetting across the delay of 1 week.

In the immediate memory test, the participants claimed to have a feeling of knowing which word had been written at the tested position on about a quarter of the memory test trials. The reliability of this phenomenal memory experience was limited since the word at the tested position was truly remembered only on about half of the respective memory test trials. Thus, immediately after reading, participants showed verbatim memory with awareness (i.e., reliable explicit memory) for about 12–13% of the tested positions in the book chapter. When combining memory test responses with and without phenomenal memory awareness, participants were able to remember for about 20% of the tested positions in the book chapter exactly which word was written at that position. In the memory test after 1 week, participants claimed to have a feeling of knowing on almost none of the memory test trial, and in the rare case when having one, this phenomenal memory experience was completely unreliable. Thus, other than implicit verbatim memory without awareness, explicit verbatim memory with awareness seems to decay within a delay of 1 week.

Interestingly, the size of the observed memory effect was in the range observed in studies demonstrating the existence of long-lasting detailed long-term memory representations for incidentally encoded non-verbal stimuli such as pictures or sounds (Kuhbandner et al., 2017; Hutmacher and Kuhbandner, 2020). When evaluating the size of the observed memory effect, it is important to note that only memory for words was tested that were theme-unrelated and non-central. It is likely that memory rates are higher when verbatim memory for theme-related and more central words is tested. For instance, as shown in the above-mentioned study on memory for unattended and incidentally encoded pictures (Kuhbandner et al., 2017), if a memory test places lower demands on the quality of the stored memory representations, higher memory rates are observed.

The present study supports the recent evidence that long-lasting memory representations of the surface details of texts are stored even when texts are read without any intention of learning (Gurevich et al., 2010). In particular, the present findings demonstrate that this holds even true for the reading of whole book chapters, and that not only the syntax is remembered but also the exact words that were written at specific positions in the book. The finding of successful memory retrieval despite absent subjective memory awareness is in line with findings demonstrating the phenomenon of recognition without awareness in verbal (Craik et al., 2015), visual (Voss et al., 2008), and haptic memory (Hutmacher and Kuhbandner, 2018), supporting speculations that there is a perceptual long-term memory system that operates below awareness (Johnson, 1983). In particular, the finding that performance did not decrease across 1 week supports findings that perceptual long-term-memory representations show little forgetting (Hutmacher and Kuhbandner, 2020) and may even be permanently stored (Mitchell, 2006).

Examining the amount of forgetting in more detail may be an interesting avenue for future research. First, a methodological limitation of the present study is that the tested pages were not counterbalanced across the immediate and delayed memory tests. The problem that may arise from such a test order is that if the pages tested in the initial test were actually easier to remember, this may have masked the occurrence of forgetting. Although it seems unlikely that there were any systematic differences in difficulty between the pages tested in the immediate and delayed tests, the finding that forgetting was absent for words remembered verbatim without awareness should be supported by further research. Furthermore, since the present study examined only verbatim memory for theme-unrelated and non-central words, it may also be interesting to examine whether verbatim memories for theme-related and central words versus theme-unrelated and non-central words show different patterns of forgetting, and whether verbatim and gist memories show different amounts of forgetting.

Regarding the subjective memory awareness of participants, participants were only asked to provide a judgement on whether a response in the memory tests was based on explicit memory (either recollection or familiarity; instructed response: “know”) or implicit memory (guessed without any phenomenal experience of recollection or familiarity; instructed response: “guess”). Thus, based on the data presented in this study, it cannot be determined whether the explicit memories for the words reflect recollection (i.e., remembering contextual details such as the location in the sentence) or familiarity (i.e., phenomenal feeling of knowing without remembering any contextual details). Clarifying this question may be another interesting avenue for future research. However, since the number of “know” responses was low in the present study, especially in the delayed memory test, the memory paradigm may have to be adapted in order to clarify this question.

The present findings may also be relevant for educational contexts. When acquired knowledge is assessed with multiple-choice tests, learners may be able to reach correct answers after reading textbooks once. However, since the memories acquired through reading are not accompanied by phenomenal memory experience, such memories are not helpful to answer open questions, indicating that actually no knowledge in a true sense has been acquired. Interestingly, this deviates from the abilities of savants like Kim Peek who was able to retrieve his verbatim memories in response to open questions. Thus, an interesting question for future research is how implicit verbatim memories can inform performance on explicit memory tasks.

## Data Availability Statement

The datasets generated for this study are available on request to the corresponding author.

## Ethics Statement

Ethical review and approval was not required for the study on human participants in accordance with the local legislation and institutional requirements. The patients/participants provided their written informed consent to participate in this study.

## Author Contributions

The author confirms being the sole contributor of this work and has approved it for publication.

### Conflict of Interest

The author declares that the research was conducted in the absence of any commercial or financial relationships that could be construed as a potential conflict of interest.

## Footnotes

^1^https://osf.io/e6a97
